# Glutamine Supplementation Prevents Chronic Stress-Induced Mild Cognitive Impairment

**DOI:** 10.3390/nu12040910

**Published:** 2020-03-26

**Authors:** Ji Hyeong Baek, Soonwoong Jung, Hyeonwi Son, Jae Soon Kang, Hyun Joon Kim

**Affiliations:** Department of Anatomy and Convergence Medical Sciences, Bio Anti-aging Medical Research Center, Institute of Health Sciences, Gyeongsang National University Medical School, Jinju 52727, Korea; birth1110@gnu.ac.kr (S.J.); hyeonwi.son@gmail.com (H.S.); jskang@gnu.ac.kr (J.S.K.)

**Keywords:** chronic stress, mild cognitive impairment, glutamine, oxidative stress

## Abstract

We recently reported that glutamine (Gln) supplementation protected glutamatergic neurotransmission from the harmful effects of chronic stress. Altered glutamatergic neurotransmission is one of the main causes of cognitive disorders. However, the cognitive enhancer function of Gln has not been clearly demonstrated thus far. Here, we evaluated whether and how Gln supplementation actually affects chronic stress-induced cognitive impairment. Using a chronic immobilization stress (CIS) mouse model, we confirmed that chronic stress induced mild cognitive impairment (MCI) and neuronal damage in the hippocampus. In contrast, Gln-supplemented mice did not show evidence of MCI. To investigate possible underlying mechanisms, we confirmed that CIS increased plasma corticosterone levels as well as brain and plasma levels of reactive oxygen/nitrogen species. CIS also increased levels of inducible nitric oxide synthase and NADPH oxidase subunits (p47^phox^ and p67^phox^) in both the prefrontal cortex and CA1 region of the hippocampus. CIS decreased the number of synaptic puncta in the prefrontal cortex and hippocampus, but these effects were inhibited by Gln supplementation. Taken together, the present results suggest that Gln is an effective agent against chronic stress-induced MCI.

## 1. Introduction

Mild cognitive impairment (MCI) represents a mental stage between normal aging and dementia. It involves problems with memory, language, clarity of thinking, and judgment, without interfering with daily life and common activities [1]. MCI has been reported to increase the risk of developing dementia caused by Alzheimer’s disease (AD) and other neurological disorders [1,2]. Therefore, the early treatment of MCI is of increasing focus, before it advances to fully developed dementia [1,2]. 

Emerging evidence has shown that disturbed glutamatergic neurotransmission in the central nervous system is one of the main causes of emotional and cognitive disorders [3,4,5,6]. As glutamate (Glu) is the most important excitatory neurotransmitter, and glutamine (Gln) is its precursor, Glu release and extracellular levels of both Glu and Gln must be strictly regulated to ensure optimal neurotransmission and normal brain function [3]. Inhibition of Glu and Gln synthesis, their release, their clearance, their metabolism, and/or their receptors can cause impaired glutamatergic signaling and cognitive dysfunction [5,7,8,9,10,11]. We recently reported that reduced glutamine synthetase activity during synaptogenesis resulted in a spatial memory impairment in adult mice and in reductions in both synaptic puncta and glutamatergic neurotransmission in the hippocampus [10].

Excessive oxidative stress contributes to neuronal damage during aging and in neurological disorders, and is linked to cognitive impairments such as dementia, amyotrophic lateral sclerosis, and Parkinson’s disease [12,13]. Moreover, it is widely accepted that chronic oxidative stress and inflammation are major pathogenic features of cognitive dysfunction linked to abnormal glutamatergic neurotransmission [13,14]. Intense or long-lasting oxidative stress leads to cellular damage by lipid peroxidation, DNA modification, inflammation, and enzyme inactivation [12]. 

Our previous studies show that Gln is a key mediator of astrocyte–neuron interactions for glutamatergic signaling, and that the direct infusion of Gln into mice increased both Glu and Gln levels in the prefrontal cortex (PFC) as well as enhanced glutamatergic activity [4,6]. Furthermore, we showed that dietary Gln supplementation prevented chronic stress-induced decreases in Glu and Gln levels, their transporter protein levels, and overall glutamatergic signaling in mouse PFC [4,15,16]. Gln has also been reported to be neuroprotective against oxidative stress and inflammation [17]. These results suggest that Gln might protect the brain from chronic stress-induced MCI. However, the cognitive enhancer effect of Gln in a MCI model and its mechanism have not been demonstrated thus far. We therefore tested dietary Gln supplementation for its ability to protect cognition from chronic stress. We also investigated the action mechanism of Gln on MCI by evaluating the effects of Gln on oxidative stress, which is one of the major cause of cognitive impairment. 

## 2. Materials and Methods 

### 2.1. Animals

Male 7-week-old C57BL/6 mice (Koatech, Pyeongtaek, Republic of Korea) were habituated for 1 week prior to experiments in a specific-pathogen-free animal facility at the School of Medicine, Gyeongsang National University. Mice were individually housed at a constant temperature (22–24 °C) under a 12 h light/dark cycle (lights on at 18:00) with free access to laboratory chow and water. A Gln-supplemented diet (150 mg/kg chow) was fed to the Gln group, and normal chow was fed to the control group (Uni Faith, Seoul, Republic of Korea) during the entire experimental period, as previously described [4,15]. Mice were randomly assigned to a group using a computer-generated list according to body weight. Control (CTL) and CTL+Gln groups were caged without or with Gln supplementation, respectively. Stress (STR) and STR+Gln groups were exposed to stress without or with Gln supplementation, respectively. Animal use procedures were performed in accordance with the National Institutes of Health guidelines and an approved protocol (GNU-161128-M0068) by the Gyeongsang National University Institutional Animal Care and Use Committee. 

### 2.2. Chronic Immobilization Stress Regimen

**Figure 1:**
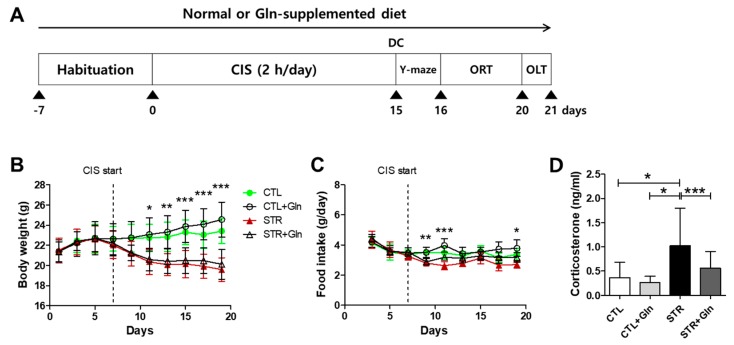
Chronic immobilization stress (CIS) induces stress responses, and glutamine (Gln) supplementation acts as an anti-stress agent. (**A**) Experimental schedule. Two cohorts were sacrificed after CIS for molecular analyses, and the other was used for behavioral testing. Body weight (**B**) and food intake (**C**) were recorded every 2 days for 3 weeks from day 0. (**D**) The effects of immobilization stress and Gln on plasma corticosterone levels. The bars charts represent means ± SDs. * *p* < 0.05, ** *p* < 0.01, and *** *p* < 0.001 values in a two-way analysis of variance (ANOVA) followed by Bonferroni post-tests (**B**,**C**) or one-way ANOVA followed by Newman–Keuls multiple-comparison tests (**D**). CTL, control (*n* = 13); CTL+Gln, Gln-supplemented control (*n* = 13); DC, decapitation; ORT, object recognition test; OLT, object location memory test; STR, stressed (*n* = 12); STR+Gln, Gln-supplemented stressed (*n* = 13).

CIS was carried out as previously described [4,15]. Briefly, mice were repeatedly placed in a restrainer for 2 h/day (14:00–16:00) for 15 days under 200 lux light conditions. Body weight and food intake were measured every other day (Figure 1A). After CIS, one cohort of mice was subjected to behavioral tests for depression (*n* = 7 per experimental group, total 28 mice). Another cohort was sacrificed by decapitation between 21:00–23:00 on the following day of the last stress session without behavioral testing (*n* = 12–13 per experimental group, total 51 mice). This cohort was used for molecular analyses of plasma corticosterone levels, reactive oxygen/nitrogen species (ROS/RNS), and protein expression levels. The other cohort was perfused with tissue fixative without behavioral testing for immunohistochemistry and Nissl staining (*n* = 8 per experimental group, total 32 mice). 

### 2.3. Y-Maze Test

The Y-maze apparatus consisted of three opaque arms, 120° apart, designated as A, B, and C. The dimensions of each arm was 40 × 8 × 15 cm. The day following the last CIS session, the Y-maze test was performed to evaluate exploratory behavior and memory. Mice were brought to the test room in home cages 30 min before the test to minimize any movement-related stress for all behavioral tests. Each mouse was placed in an arm (always the B arm), facing opposite the center of the maze and was allowed to move through the apparatus for 8 min. Entry into an arm was only considered complete when all four paws were inside of it. The first entry (B) was excluded from calculations. Spontaneous alternation (expressed as a percentage) was defined as the number of consecutive entries into the three different arms, divided by the total number of possible triads (total arm entries − 2) [18] according to the formula:

(1) $$\frac{\mathrm{Number of spontaneous alternations}}{\left(\mathrm{total arm entries}\;-\;2\right)}\times 100,$$

### 2.4. Object Recognition Test (ORT) and Object Location Memory Test (OLT)

To investigate recognition memory, an ORT and an OLT were both performed, as previously described, with slight modifications [19,20]. The ORT consisted of three sessions: habituation, familiarization, and a test session. An open-field box with 1 cm bedding was used. During habituation, each mouse was placed in the empty open field for 10 min a day for 2 days. After habituation, familiarization was conducted by placing two identical objects at two positions within the open field. Each mouse was placed in the open field with its nose opposite the objects and was allowed to freely explore for 10 min. The next day, one of the objects was replaced with a novel object, and the test was repeated. Only the first 5 min of each session was used for analysis. The discrimination index (DI) for the ORT was calculated as follows:

(2) $$\frac{\left(\mathrm{time exploring the novel object}\;-\;\mathrm{time exploring the familiar}\right)}{\left(\mathrm{time exploring the novel}\;+\;\mathrm{the familiar}\right)},$$

A DI of zero indicates equal preference for the two objects, and a large negative DI could indicate neophobia of the novel object. 

The day after the ORT, the OLT was conducted in the same open field. The same novel object of the ORT was moved to a different position in the field, and each mouse was allowed to explore the objects for 5 min. The DI for the OLT was calculated as follows:

(3) $$\frac{\left(\mathrm{time exploring the moved object}\;-\;\mathrm{time exploring the familiar}\right)}{\left(\mathrm{time exploring the moved}\;+\;\mathrm{the familiar}\right)},$$

### 2.5. Enzyme-Linked Immunosorbent Assay (EIA) of Plasma Corticosterone

Mouse blood was collected in vacutainers containing K_3_EDTA. Plasma was isolated by centrifugation at 1000 × *g*, at 4 °C for 10 min. The samples were stored at −80 °C until use. Quantification of plasma corticosterone (CORT) levels was carried out using a CORT enzyme-linked immunosorbent assay kit (Cayman, Ann Arbor, MI, USA), according to the manufacturer’s protocol. The absorbance at 412 nm was measured using a Tecan Infinite F200 PRO microplate reader (Tecan Austria GmbH, Männedorf, Austria). Each sample was tested in triplicate.

### 2.6. Total ROS/RNS Assay

The total ROS/RNS levels in plasma and tissue samples were measured using an OxiSelect ROS/RNS assay kit (STA-347; Cell Biolabs, San Diego, CA, USA), according to the manufacturer’s instructions. Tissues (PFC and hippocampus) were lysed by homogenization with glass beads in radioimmunoprecipitation assay (RIPA) buffer (Elpis-Biotech, Daejeon, Republic of Korea) using a Bullet Blender (Next Advance, New York, NY, USA), followed by sonication for 2 min and centrifugation at 12,000 × *g*, at 4 °C for 10 min. Fluorescence intensity was measured using a Tecan Infinite F200 PRO microplate reader (Tecan Austria GmbH). Each sample was tested in triplicate.

### 2.7. Immunohistochemistry (IHC)

IHC was performed as previously described [4], with some modifications. Twenty-four hours after the last stress session, mice were deeply anesthetized with avertin and perfused with phosphate-buffered saline (pH 7.4) and 4% paraformaldehyde. The brains were collected, postfixed, sectioned at a thickness of 40 μm, and incubated at 4 °C overnight with the following antibodies for: nNOS (sc-5302, 200 µg/mL, Santa Cruz, Dallas, TX, USA, 1:50), iNOS (sc-7271, 200 µg/mL, Santa Cruz, 1:20), p47^phox^ (sc-17845, 200 µg/mL, Santa Cruz, 1:20), p67^phox^ (15551-1-AP, 43 μg/150 μL, Proteintech, Rosemont, IL, USA, 1:100), postsynaptic density (PSD)-95 (ab12093, 1 mg/mL, Abcam, Cambridge, UK, 1:200), and synaptophysin (ab14692, 0.65 mg/mL, Abcam, 1:200). Ionized calcium-binding adapter molecule 1 (Iba1; ab5076, 0.5 mg/mL, Abcam, 1:200), neuronal nuclei (NeuN; MAB377, 30 μg/mL, Merck Millipore, St. Louis, MO, USA, 1:200), and glutamate decarboxylase (GAD2; #3988, Cell Signaling, Danvers, MA, USA, 1:100) were used as cellular distribution markers. Brain slices were then incubated with AlexaFluor 594- and 488-conjugated secondary antibodies (2 mg/mL, Invitrogen, Carlsbad, CA, USA, 1:1000). Secondary antibody-only controls were performed to confirm specific binding of the primary antibodies. Digital images were captured using a spinning-disk confocal microscope equipped with an Olympus Disk Spinning Unit (Olympus, Tokyo, Japan) and analyzed using ImageJ software (NIH). Eight mice per group (total 32 mice) were used for brain section preparation. Two images were captured from one section at the same region in the right and left hemispheres. Therefore, sample number for statistical analysis was 16/group. 

### 2.8. Nissl Staining

Mounted and air-dried sections were stained in 0.1% Cresyl Violet (in 0.2% acetic acid) for 5 min. Sections were washed and dehydrated in ethanol: 80% for 30 s, 90% for 30 s, 95% for 30 s twice, and 100% for 30 s twice. Sections were cleared in xylen for 1 min twice, covered with xylen mounting solution (xylen:Permount = 1:1) (Permount, Fisher Chemical, Hampton, NH, USA), and then dried in the fume hood. 

### 2.9. Western Blot Analysis

Protein samples (10 μg) were separated by sodium dodecyl sulfate-polyacrylamide gel electrophoresis and transferred onto polyvinylidene difluoride membranes. The membranes were blocked with 5% skim milk for 2 h, incubated at 4 °C overnight with a primary antibody (1:200), and then incubated with a secondary antibody conjugated to horseradish peroxidase (1:10,000) to detect the targeted protein. Immunoblot signals were detected using an enhanced chemiluminescence detection kit (Thermo Fisher Scientific) and an iBright FL1000 (Thermo Fisher Scientific). Band intensities were calculated using iBright analysis software (Thermo Fisher Scientific). 

### 2.10. Statistical Analysis

Data are presented as the means ± standard deviations (SDs). Differences between groups were first determined using a two-way analysis of variance (ANOVA) followed by Bonferroni post-test or one-way ANOVA followed by Newman–Keuls multiple-comparison tests in GraphPad (La Jolla, CA, USA) software. The significance level was set at *p* less than 0.05. 

## 3. Results

### 3.1. CIS-Induced MCI Was Protected by Gln Supplementation

We used chronic immobilization to induce cognitive impairment in young male mice (Figure 1A), the same process used for chronic stress-induced depression in our previous studies [4,15,16,18]. As expected, CIS reduced body weight in the stressed groups (Figure 1B), without a difference in food intake (Figure 1C). CIS markedly increased plasma CORT levels in the STR group, but this increase was inhibited by Gln supplementation (Figure 1D). These results are similar to those reported in our previous studies [4,15,16,18], confirming that CIS induces a stress response, and supplementation with Gln blunts its effects.

**Figure 2:**
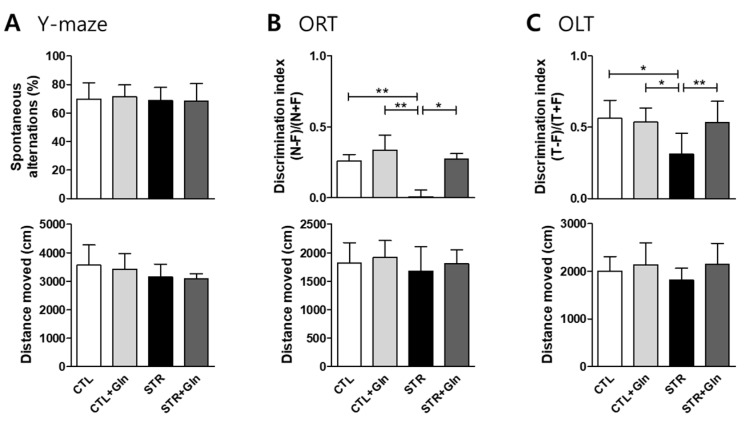
The harmful effects of chronic immobilization stress (CIS) on learning and memory are blocked by glutamine (Gln) supplementation. Short-term working memory was evaluated in mice using spontaneous alternations in a Y-maze (**A**), and learning and memory was evaluated using an object recognition test (**B**; ORT) and an object location memory test (**C**; OLT). The bar charts represent means ± SDs. * *p* < 0.05 and ** *p* < 0.01 values in a one-way analysis of variance followed by Newman–Keuls multiple-comparison tests (*n* = 7/group, **A**–**C**). CTL, control; CTL+Gln, Gln-supplemented control; STR, stressed; STR+Gln, Gln-supplemented stressed.

To evaluate CIS-induced MCI in these mice, we measured short-term working memory function using Y-maze spontaneous alternations, and learning and memory functions for objects and spatial recognition using an ORT and an OLT, respectively. Short-term working memory was not affected by CIS (Figure 2A) but learning and memory functions for objects (Figure 2B) and object locations (Figure 2C) were significantly decreased in the STR group. These deleterious effects of CIS on learning and memory were ameliorated by Gln supplementation (Figure 2B,C). There was no significant difference in motor activity between groups (Figure 2). 

### 3.2. Gln Supplementation Prevented CIS-Induced Morphological Changes in the Hippocampus and Increases in ROS/RNS 

**Figure 3:**
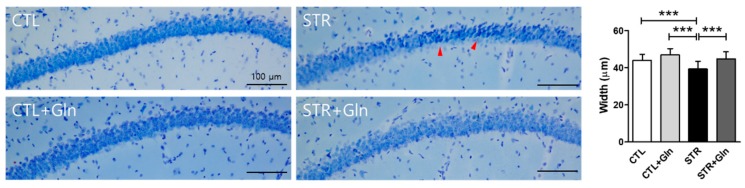
Glutamine (Gln) supplementation prevents chronic immobilization stress-induced morphological changes in the hippocampus. The width of the stratum pyramidale CA1 in the hippocampus was measured on Nissl-stained brain slides. Red arrows indicate small and darkly stained cells. Scale bars = 100 μm. The bar charts represent means ± SDs. *** *p* < 0.001 values in a one-way analysis of variance followed by Newman–Keuls multiple-comparison tests (*n* = 16/group). CTL, control; CTL+Gln, Gln-supplemented control; STR, stressed; STR+Gln, Gln-supplemented stressed.

The width of the stratum pyramidale CA1 (CA1-SP) in the hippocampus of the STR group was reduced compared to that of the CTL group (Figure 3). In addition, neurons in the CA1-SP of the STR group appeared smaller, more darkly stained, and had indistinct Nissl bodies, indicating a similarity between CIS-induced hippocampal neuron damage and that reported in patients with MCI or AD [19,20]. However, Gln supplementation prevented these anatomical changes in the hippocampus: The CA1-SP width in the STR+Gln group was no different from the CTL width after CIS, and lacked the smaller, more darkly stained neurons observed in the STR group. The PFC did not show obvious morphological changes due to either CIS or Gln supplementation (data not shown). 

**Figure 4:**
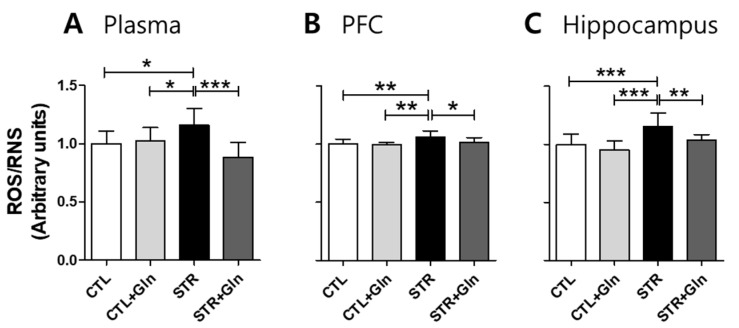
Glutamine (Gln) supplementation prevented chronic immobilization stress-induced reactive oxygen/nitrogen species (ROS/RNS) increase. Relative ROS/RNS levels in (**A**) plasma, (**B**) prefrontal cortex (PFC), and (**C**) hippocampus. The bar charts represent means ± SDs (arbitrary units). * *p* < 0.05, ** *p* < 0.01, and *** *p* < 0.001 values in a one-way analysis of variance followed by Newman–Keuls multiple-comparison tests. CTL, control (*n* = 13); CTL+Gln, Gln-supplemented control (*n* = 13); STR, stressed (*n* = 12); STR+Gln, Gln-supplemented stressed (*n* = 13).

We also investigated chronic stress-induced total ROS/RNS accumulation in the plasma, PFC, and hippocampus (Figure 4). Total ROS/RNS levels increased due to CIS in the plasma, PFC, and hippocampus in the STR group, but Gln supplementation blocked these stress-induced changes in the STR+Gln group. 

### 3.3. Gln Prevented CIS-Induced iNOS and NOX Expression, and Maintained Synaptic Puncta in the PFC and Hippocampus during CIS

**Figure 5:**
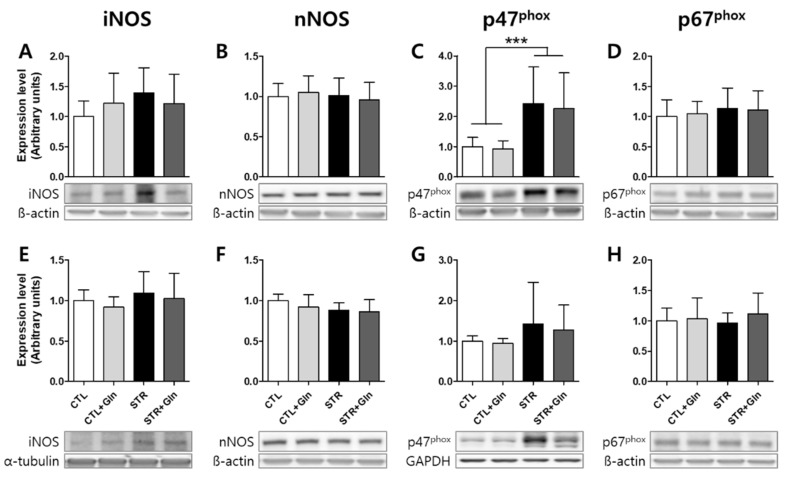
Protein expression changes related to reactive oxygen/nitrogen species (ROS/RNS) evaluated by immunoblotting. (**A**–**D**) Prefrontal cortex (PFC) and (**E**–**H**) hippocampus. The bar charts represent means ± SDs (arbitrary units). *** *p* < 0.001 value in a one-way analysis of variance followed by Newman–Keuls multiple-comparison tests. CTL, control (*n* = 13); CTL+Gln, Gln-supplemented control (*n* = 13); iNOS, inducible nitric oxide synthase; nNOS, neuronal NOS; p47^phox^ and p67^phox^ are NADPH oxidase cytosolic subunits; STR, stressed (*n* = 12); STR+Gln, Gln-supplemented stressed (*n* = 13).

**Figure 6:**
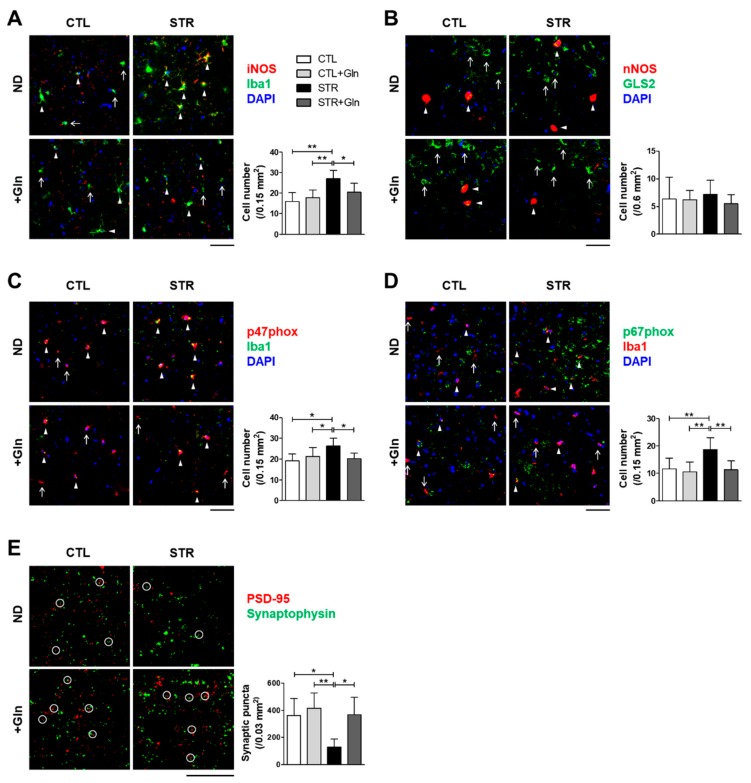
Glutamine (Gln) supplementation prevents both the increase in proteins related to chronic immobilization stress-induced reactive oxygen/nitrogen species (ROS/RNS), and the reduction of synaptic puncta in the infralimbic cortex. (**A**–**D**) Representative images of infralimbic cortex immunohistochemistry of ROS/RNS production-related proteins with staining for microglial marker Iba1. Arrowheads indicate double-positive cells and arrows indicate cellular marker single-positive cells. Double-positive cell numbers are presented with the images. (**E**) Numbers of synaptic puncta colocalized with PSD-95 and synaptophysin (white circles) in the infralimbic cortex are presented with representative images. Secondary antibody-only controls were performed to confirm specific binding of the primary antibodies (data not shown). * *p* < 0.05 and ** *p* < 0.01 values in a one-way analysis of variance followed by Newman–Keuls multiple-comparison tests (*n* = 16/group, **A**–**E**). Scale bars = 50 μm. CTL, control; CTL+Gln, Gln-supplemented control; STR, stressed; STR+Gln, Gln-supplemented stressed; ND, normal diet.

**Figure 7:**
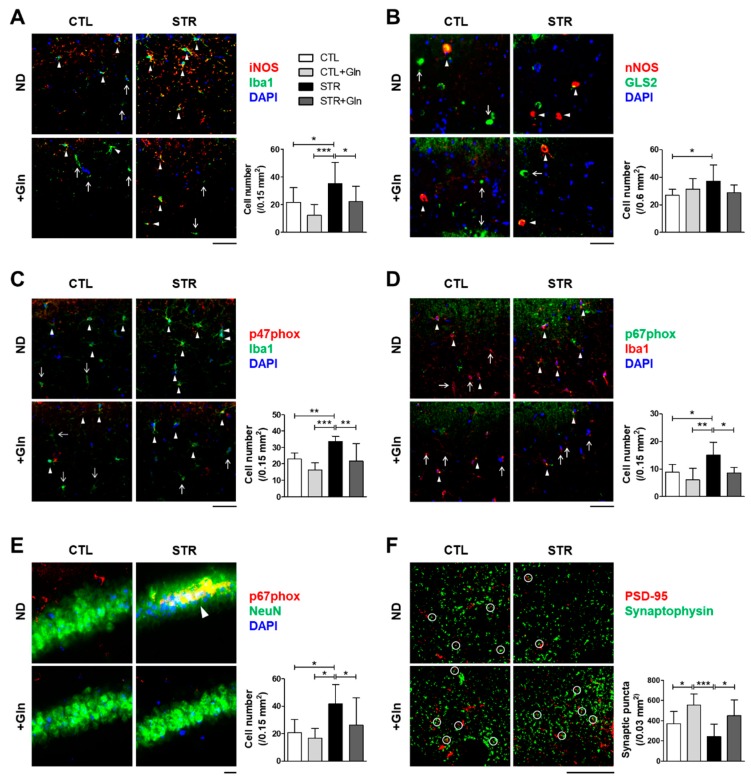
Glutamine (Gln) supplementation prevents both the increase in proteins related to chronic immobilization stress-induced reactive oxygen/nitrogen species (ROS/RNS), and the reduction of synaptic puncta in the hippocampal CA1 region. (**A**–**E**) Representative images of hippocampal immunohistochemistry for ROS/RNS production-related proteins with cellular markers (Iba1 for microglia and NeuN for neurons). Images were analyzed in the stratum radiatum (**A**–**D**) or the stratum pyramidale (**E**) in the CA1 region. Arrowheads indicate double-positive cells and arrows indicate cellular marker single-positive cells. Double-positive cell numbers are presented with the images. (**F**) Numbers of synaptic puncta colocalized with PSD-95 and synaptophysin (white circles) in the stratum radiatum CA1 are presented with representative images. Secondary antibody-only controls were performed to confirm specific binding of the primary antibodies (data not shown). * *p* < 0.05, ** *p* < 0.01, and *** *p* < 0.001 values in a one-way analysis of variance followed by Newman–Keuls multiple-comparison tests (*n* = 16/group, **A**–**F**). Scale bars = 50 μm. CTL, control; CTL+Gln, Gln-supplemented control; STR, stressed; STR+Gln, Gln-supplemented stressed; ND, normal diet.

To investigate how Gln affects ROS/RNS levels, ROS/RNS production-related proteins were evaluated by immunoblotting and IHC (Figure 5, Figure 6 and Figure 7). We examined the PFC and hippocampal expression levels of inducible nitric oxide synthase (iNOS), neuronal NOS (nNOS), and two nicotinamide adenosine dinucleotide phosphate oxidase (NOX) cytosolic subunits: p47^phox^ and p67^phox^.

iNOS and p47^phox^ seemed to be increased in the STR group, but not in the STR+Gln group, although there was no statistical significance in the immunoblotting results using crude protein extracts (Figure 5). Crude tissue lysates may not reflect cell-type-specific protein expressional changes due to surrounding non-responsive cells. To clarify these changes in expression, IHC was performed in both the PFC and hippocampus (Figure 6 and Figure 7). The immunostaining for iNOS and the NOX subunits (p47^phox^ and p67^phox^) was significantly increased by CIS in microglia (colocalized using Iba1 staining) within the infralimbic cortex (Figure 6) and in the hippocampal CA1 region (Figure 7). p67^phox^ immunostaining was also increased in pyramidal neurons of the CA1 region (Figure 7E). Gln supplementation prevented the induction of all of these proteins, supporting the systematic changes seen in ROS/RNS levels by CIS and after Gln supplementation (Figure 4). Changes in nNOS expression, by either CIS or Gln supplementation, were not significant in either the PFC or the hippocampus (Figure 6B and Figure 7B).

To evaluate the effects of oxidative stress on synaptic plasticity, synaptic contacts (determined by the colocalization of immunostaining for PSD-95 and synaptophysin) were counted in the PFC and hippocampus CA1 region. The number of synaptic puncta in the PFC was reduced in the STR group but not in the STR+Gln group (Figure 6E). CIS decreased synaptic puncta in the hippocampal CA1 region, but this finding was not statistically significant (Figure 7F). In contrast, Glu supplementation significantly increased synaptic puncta in the CA1 regions of both CTL+Gln and STR+Gln groups compared to the non-Gln supplemented groups (CTL and STR; Figure 7F). These results suggest that Gln supplementation maintains functional synapses in the PFC and increases synaptic puncta in the hippocampus during chronic stress. 

## 4. Discussion

We induced MCI in mice using a CIS regimen, a model used for chronic stress-induced depression in our previous studies [4,15,16,18]. It is well known that chronic stress is a main cause of cognitive impairment and early onset of dementia [3,21,22]. As expected, this CIS regimen evoked mild cognitive impairment with increased plasma levels of the stress hormone CORT (Figure 1 and Figure 2). Moreover, this CIS regimen induced neural damage in the hippocampus (Figure 3) without severe neuronal death. These changes confirm that our mouse CIS regimen is a suitable model for MCI research. 

We also found that a Gln-supplemented diet could serve as an anti-stress agent by preventing CIS-induced MCI in mice (Figure 1 and Figure 2). Cognitive impairment induced by chronic stress might be mediated via the harmful effects of glucocorticoids and oxidative stress [3,23,24]. High levels of glucocorticoids, caused by repeated stress, can alter receptor-possessing cells and trigger oxidative stress responses in different brain regions, including the hippocampus and the PFC [24,25,26]. As shown in our previous studies [4,15,16], a Gln-supplemented diet reversed elevated blood CORT levels back to baseline. This was the impetus for investigating Gln supplementation in stress-induced MCI, and Figure 1 shows that Gln also reversed elevated blood CORT levels back to CTL levels. 

The morphological changes in the CA1 region of the STR group (Figure 3) might be due to the high levels of CORT and oxidative stress evoked by ROS/RNS (Figure 4). Neuronal cell shrinkage and pyknosis are prominent features of MCI [27,28]. It is also well known that the CA1 region is very vulnerable to oxidative stress and plays an important role in learning and memory [19,20,29]. In this study, we found that CA1 damage resulted in learning and memory problems related to both object and location recognition, but not normal short-term working memory (Figure 2). These results suggest that normal function in the CA1 region is essential for learning and memory in mice.

CIS can lead to chronic oxidative stress and inflammation, which are known to contribute to neuronal damage and cognitive dysfunction in neurological disorders such as MCI and AD [30,31,32,33]. Chronic oxidative stress and/or inflammatory responses are mainly due to long-term increases in ROS/RNS. In mammalian cells, iNOS and NOX play major roles in the production of nitric oxide (^•^NO) and superoxide (O_2_^•−^), respectively [34]. Increased nitric oxide and superoxide levels can evoke inflammatory responses in the peripheral and central nervous systems, and are well known to influence neural plasticity [12,35]. Here, we found increases in ROS/RNS in the blood, PFC, and hippocampus of STR mice (Figure 4). Accordingly, immunoblots and IHC were used to analyze the enzymes responsible for ROS/RNS production (Figure 5, Figure 6 and Figure 7). As shown in Figure 5, we could not determine any CIS-induced changes in expression using crude protein extracts from PFC or the hippocampus. However, using IHC, we found higher expression levels for these enzymes in the STR group compared with the CTL group, which is consistent with other reports [31,33,34], and we also confirmed that Gln-supplementation reversed these increases back to levels observed in the CTL group (Figure 6 and Figure 7). Therefore, the data suggest that these increased enzyme expression levels may contribute to increased ROS/RNS levels (Figure 4) and that the addition of Gln to the diet may prevent these stress-induced increases. 

As stated previously, it is widely accepted that nitric oxide and superoxide are linked to cognitive impairment and effects on neuronal plasticity in other experimental conditions and clinical cases [12,13]. Emerging evidence suggests that normal glutamatergic activity and synaptic connections are required for normal cognitive function [5,8]. We recently showed that a lower frequency of spontaneous excitatory postsynaptic currents and reduced synaptic puncta in the hippocampus cause a spatial memory impairment [10]. Therefore, we examined synaptic puncta using IHC for both PSD-95 and synaptophysin, and found that puncta were decreased in both the PFC and CA1 region of the STR group but not in STR+Gln group (Figure 6 and Figure 7). This result may be related to cognitive function in the STR group, because very early in mild AD and MCI, the total number of synapses decreases in the CA1-SR region compared to normal conditions [29].

Gln is the most abundant amino acid in the central nervous system and is a precursor of the excitatory neurotransmitter Glu [36]. Under normal conditions, Gln is synthesized in the brain via the Krebs or Glu–Gln cycle [36]. However, Gln becomes essential in organs weakened by stress, surgery or injury, and so it can also be classified as a ‘conditionally essential’ amino acid [17]. Gln has therefore been used clinically to protect tissues under stress from chemotherapy, radiation, and surgery-related catabolism by inhibiting nitric oxide production [37,38]. These studies established the clinical efficacy and safety of Gln for stress treatment. As Gln is also involved in the synthesis of glutathione, a key factor to cope with oxidative stress, deprivation of Gln or glutathione has been shown to increase ROS levels [38,39]. The cognitive maintenance function of Gln in this study may be a result of its inhibition of ROS/RNS production and subsequent neuronal damage, as well as its protective effects on glutamatergic neuronal signaling determined in our previous study [4]. 

In conclusion, we have demonstrated that Gln protects the PFC and hippocampus against CIS-induced oxidative damage by inhibiting NOS and NOX production, ultimately suppressing MCI development. Our findings suggest that Gln is a good nutraceutical candidate for preventing MCI from stress.
